# Author Correction: Genome-wide meta-analysis of phytosterols reveals five novel loci and a detrimental effect on coronary atherosclerosis

**DOI:** 10.1038/s41467-022-28863-y

**Published:** 2022-02-25

**Authors:** Markus Scholz, Katrin Horn, Janne Pott, Arnd Gross, Marcus E. Kleber, Graciela E. Delgado, Pashupati Prasad Mishra, Holger Kirsten, Christian Gieger, Martina Müller-Nurasyid, Anke Tönjes, Peter Kovacs, Terho Lehtimäki, Olli Raitakari, Mika Kähönen, Helena Gylling, Ronny Baber, Berend Isermann, Michael Stumvoll, Markus Loeffler, Winfried März, Thomas Meitinger, Annette Peters, Joachim Thiery, Daniel Teupser, Uta Ceglarek

**Affiliations:** 1 Institute for Medical Informatics, Statistics and Epidemiology, Medical Faculty, University of Leipzig, Leipzig, Germany; 2 LIFE Research Center for Civilization Diseases, Medical Faculty, University of Leipzig, Leipzig, Germany; 3 Medical Faculty Mannheim, Vth Department of Medicine, Heidelberg University, Heidelberg, Germany; 4 Department of Clinical Chemistry, Fimlab Laboratories, and Finnish Cardiovascular Research Center - Tampere, Faculty of Medicine and Health Technology, Tampere University, Tampere, 33520 Finland; 5 Research Unit of Molecular Epidemiology, Helmholtz Zentrum München, German Research Center for Environmental Health, Munich, Germany; 6 Institute of Epidemiology, Helmholtz Zentrum München, German Research Center for Environmental Health, Munich, Germany; 7 German Center for Diabetes Research (DZD), Munich, Germany; 8 Institute of Genetic Epidemiology, Helmholtz Zentrum München—German Research Center for Environmental Health, 85764 Neuherberg, Germany; 9 Institute for Medical Information Processing, Biometry and Epidemiology, Medical Faculty, Ludwig-Maximilians University of Munich, Munich, Germany; 10 Institute of Medical Biostatistics, Epidemiology and Informatics (IMBEI), University Medical Center, Johannes Gutenberg University, 55101 Mainz, Germany; 11 Department of Internal Medicine I (Cardiology), Hospital of the Ludwig-Maximilians-University Munich, 81377 Munich, Germany; 12 Medical Department III – Endocrinology, Nephrology, Rheumatology, University Hospital Leipzig, Leipzig, Germany; 13 Department of Clinical Physiology, Tampere University Hospital, and Finnish Cardiovascular Research Center - Tampere, Faculty of Medicine and Health Technology, Tampere University, Tampere, 33521 Finland; 14 Heart and Lung Center, Cardiology, University of Helsinki and Helsinki University Hospital, Helsinki, Finland; 15 Institute for Laboratory Medicine Clinical Chemistry and Molecular Diagnostics, University Hospital Leipzig, Leipzig, Germany; 16 Synlab Academy, Synlab Holding Deutschland GmbH, Augsburg, Germany; 17 Clinical Institute of Medical and Chemical Laboratory Diagnostics, Medical University Graz, Graz, Austria; 18 Institue for Human Genetics, Technical University of Munich, Munich, Germany; 19 Institue of Epidemiology, Helmholtz Zentrum München, German Research Center for Environmental Health, Munich, Germany; 20 Insitute for Laboratory Medicine, Ludwig-Maximilians University of Munich, Munich, Germany

**Keywords:** Genetics research, Molecular medicine, Genome-wide association studies, Cardiovascular diseases

Correction to: *Nature Communications* 10.1038/s41467-021-27706-6, published online 10 January 2022.

In this article the affiliation details for Helena Gylling were incorrectly given as Department of Internal Medicine, University of Helsinki and Helsinki University Hospital, Helsinki, Finland but this should have been Heart and Lung Center, Cardiology, University of Helsinki and Helsinki University Hospital, Helsinki, Finland. The original article has been corrected.

