# Supplementary material for: Genome-wide meta-analysis of phytosterols reveals five novel loci and a detrimental effect on coronary atherosclerosis
Source: Nat Commun. 2022 Jan 10;13:143. doi: 10.1038/s41467-021-27706-6 (PMC8748632; doi:10.1038/s41467-021-27706-6)

Supplementary figures

Supplementary Figure S1 (Correlation of phytosterols and zoosterols): We present pairwise partial correlation of phytosterol traits controlling for age, sex, log(BMI), diabetes status, lipid lowering medication and study and respective hierarchical clustering. Free to esterified ratios are clearly separated as well as ratios to lanosterol.

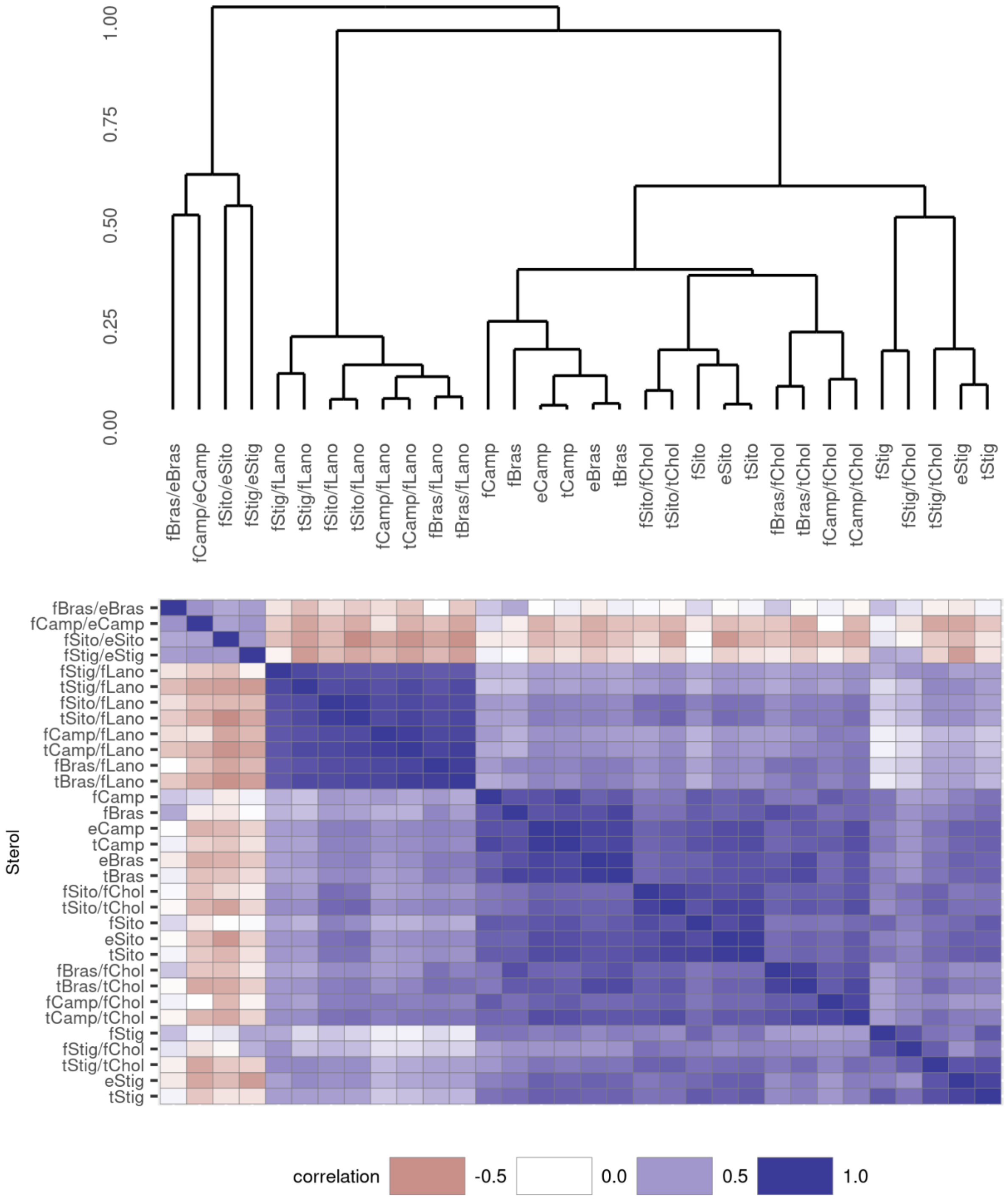

Supplementary Figure S2 (Regional association plot of conditional results of 2p21): We present regional association plots of the conditional results of the four independent variants at 2p21. A window of 100kb around the respective independent variant is presented. Dots correspond to SNP position and -log10(p-values) of fixed effect meta-analysis of gene-dose effects of total sitosterol conditioned to the three other independent variants. The large blue dot denotes the respective independent SNP of that locus. Colours of small dots indicate LD (r^2) with this SNP. We also provide annotated genes within the locus and recombination rates to mark haplo-blocks. Unconditional results are shown in figure 2 of the main document.

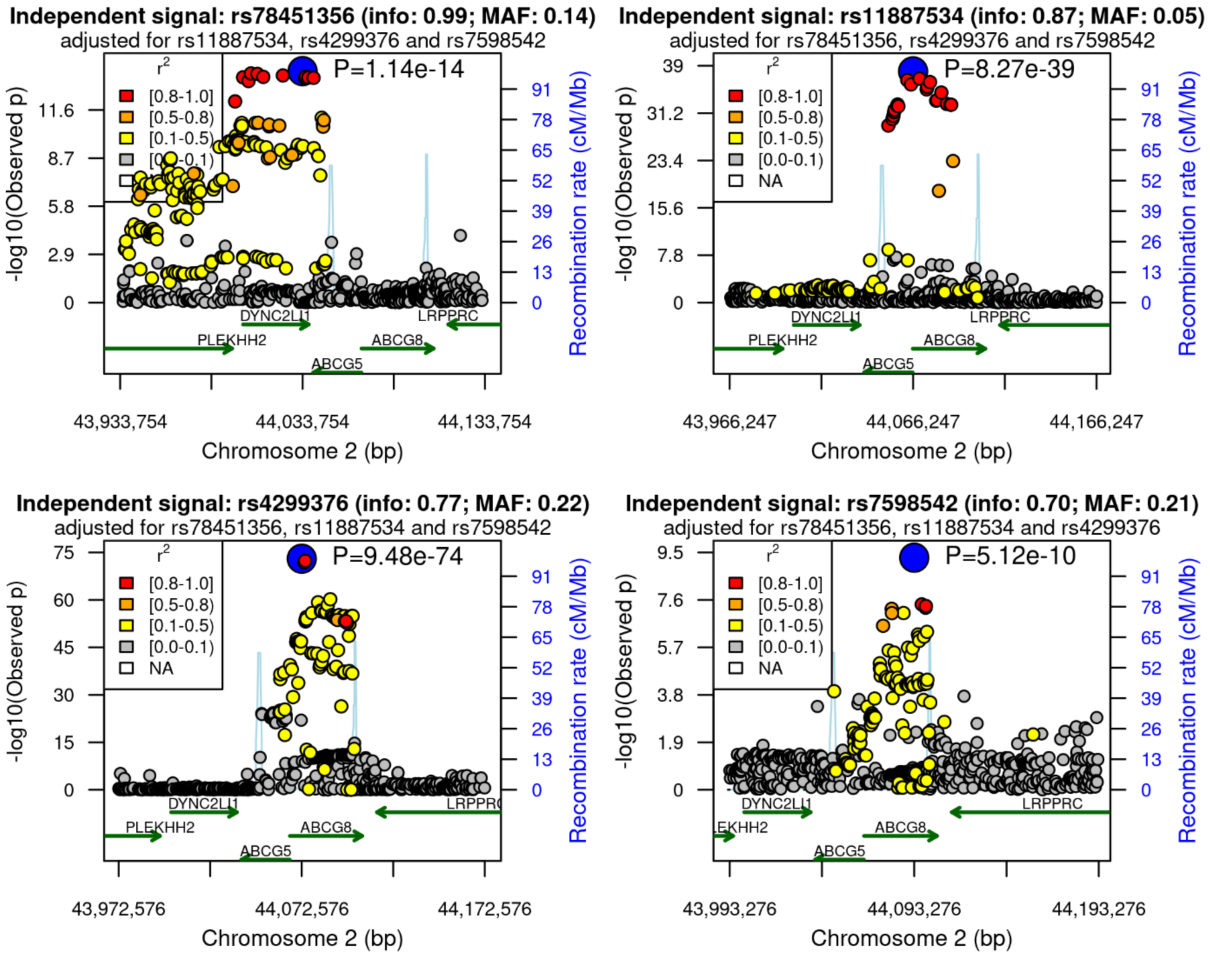

Supplementary Figure S3 (Regional association plots of other loci): Regional association plots are presented for SNPs of the six loci with only a single independent variant. A window of 100kb around the top-hit is presented. Dots correspond to SNP position and -log10(p-values) of fixed effect meta-analysis of gene-dose effects of the best associated trait at this locus. The large blue dot denotes the top-SNP of the respective locus. Colours of small dots indicate LD (r2) with the top-SNP. We also provide annotated genes within the locus and recombination rates to mark haplo-blocks. All loci are supported by other variants and could be confined to candidate genes.

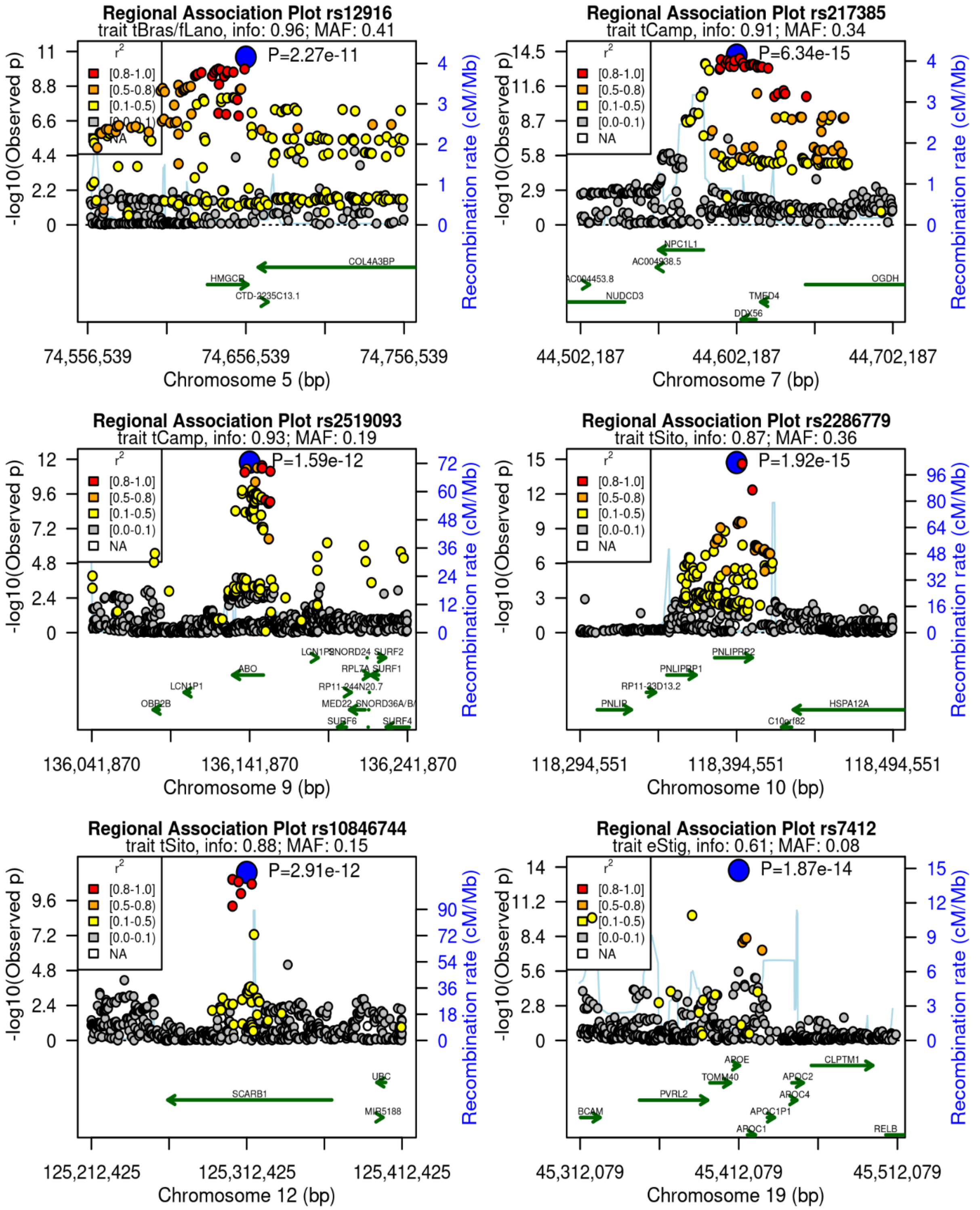

Supplementary Figure S4 (Forest plots): We present Forest plots of the ten independent SNPs identified. Best associated phenotype and corresponding test statistics are reported per study and summarized by fixed and random effect estimates. P-values are two-sided throughout. Size of symbols corresponds to weight of the study. For the four independent SNPs at 2p21, we present the unconditioned statistics. We observed direction consistency of single study results in almost all situations. Moreover, confidence intervals of single studies are typically overlapping with the meta-effect indicating low heterogeneity of single study results.

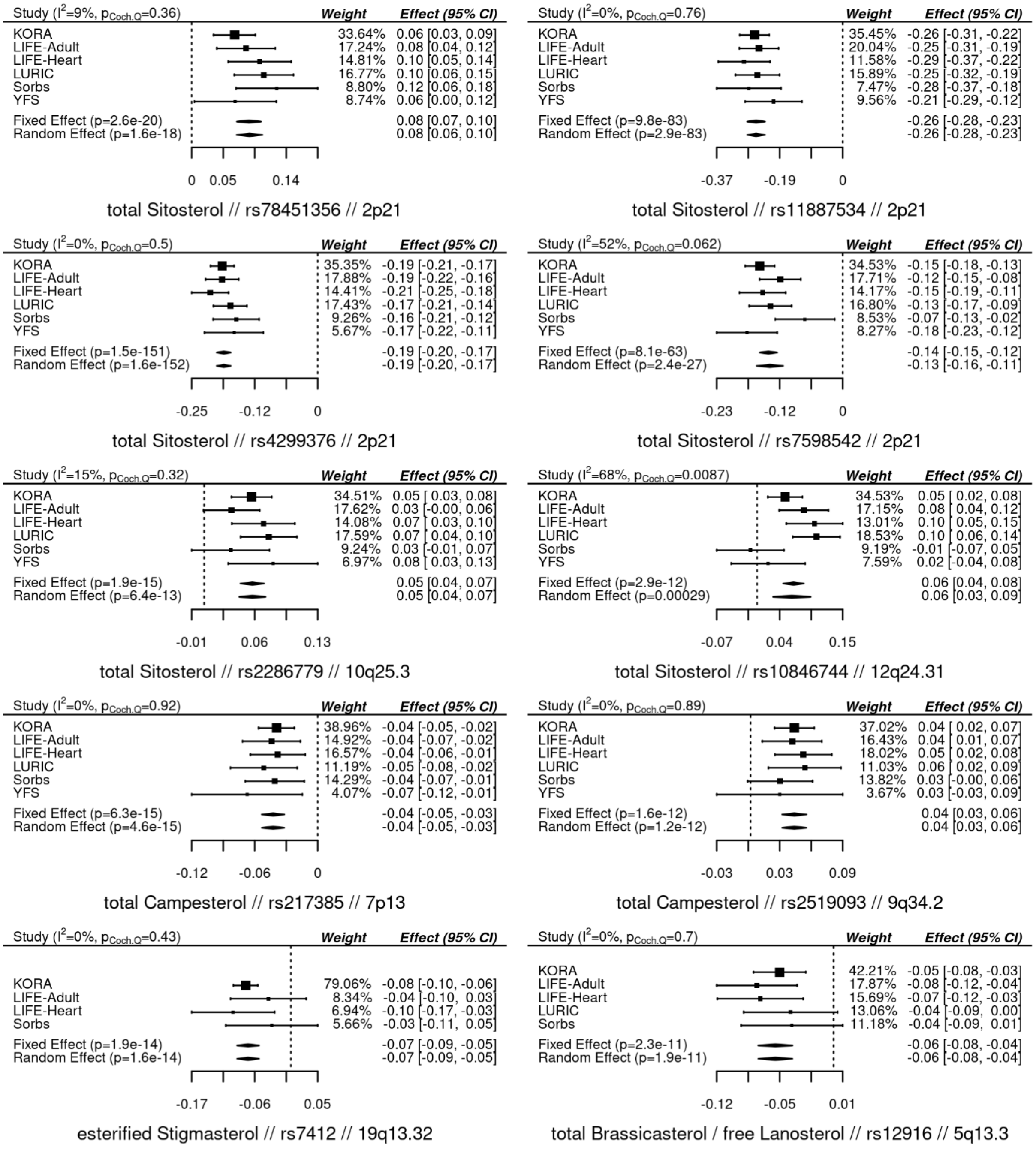

Supplementary Figure S5 (Comparisons of Beta-estimates for different genetic phytosterol associations): We compare the Beta-estimates of our top 10 loci between different classes of phytosterol traits (A: between absolute traits, B: between absolute and relative concentrations). A good agreement was observed for the absolute traits throughout. For the comparisons between absolute and relative traits, we observed larger deviations for the *ABCG5/8* locus expressing larger effect sizes for absolute concentrations and for the *HMGCR* locus which is driven by a strong lanosterol association.

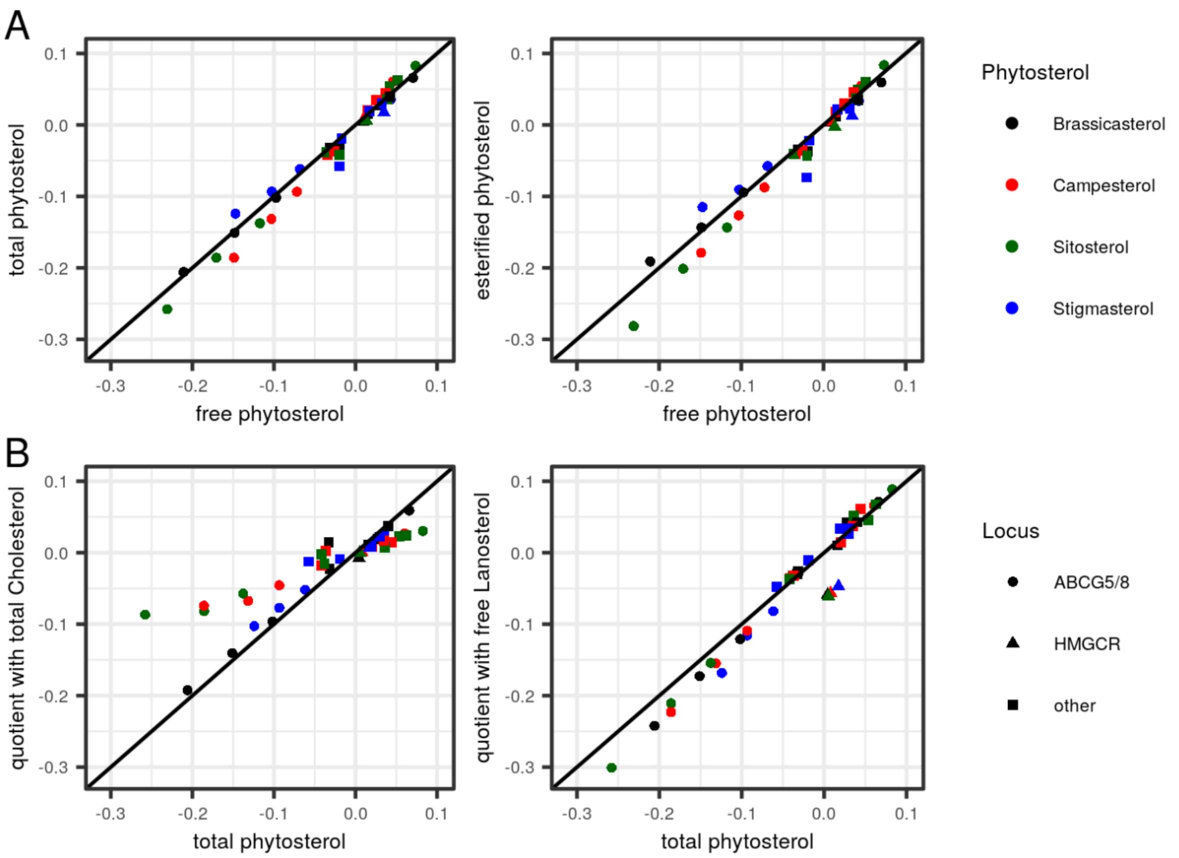

Supplementary Figure S6 (Results of eQTL Co-localization analysis): The 10 independent meta-GWAS signals were subjected to co-localization analysis with eQTLs of candidate genes shown on the left hand side of the panel and in different tissues (columns). We depict the posterior probabilities of H4 (evidence of co-localization) in blue and the negative posterior probabilities of H3 (no evidence of co-localization) in red. Darkness of colour and size of circles correspond to the numerical value of the corresponding posterior probability. Empty cells represent cases in which no eQTL signals were available. Numerical results are shown in supplemental data S9.

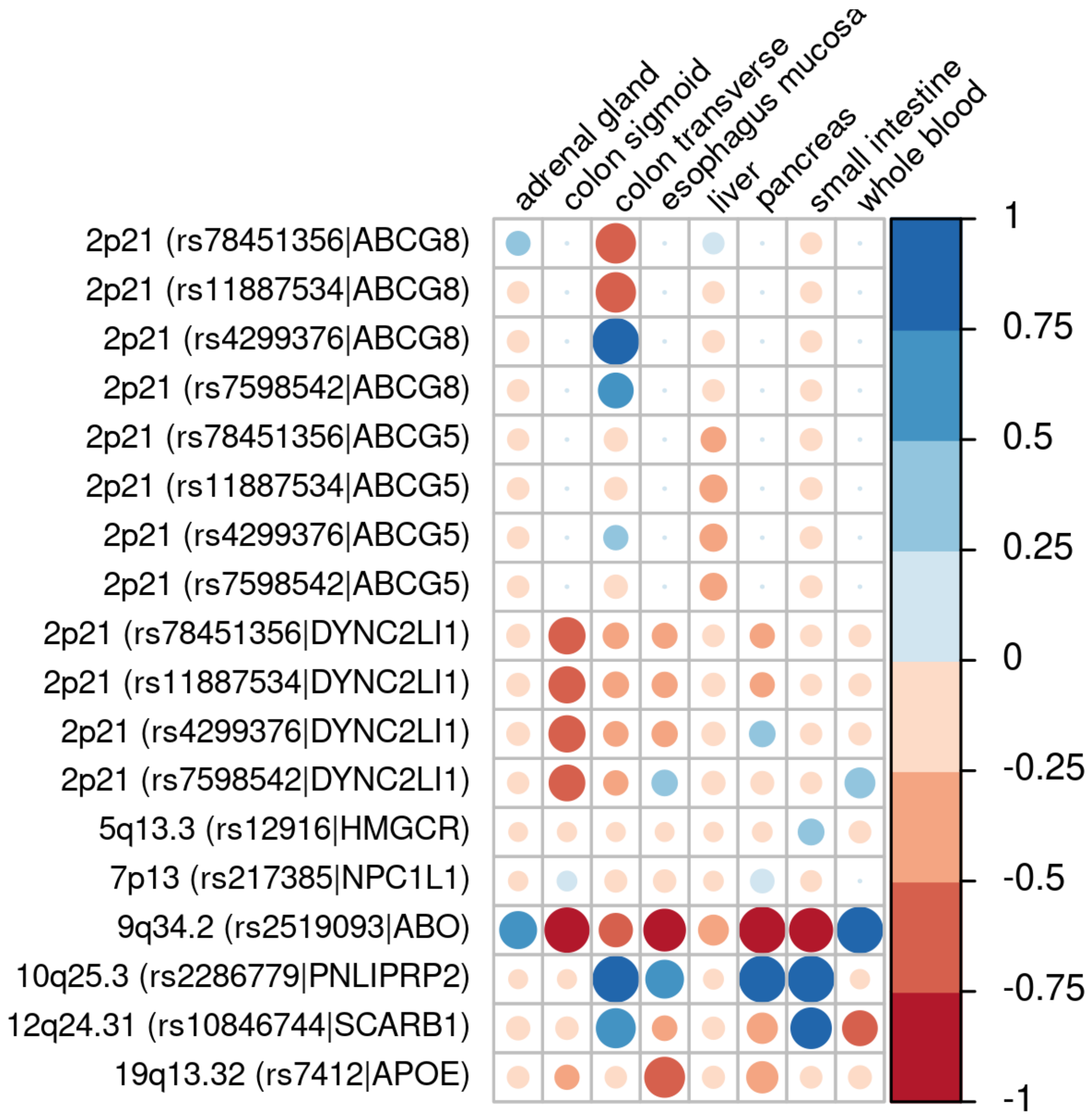

Supplementary Figure S7 (Linkage disequilibrium heatmap of ABO locus): We present position and linkage disequilibrium (Lewontin's D' at upper diagonal, r^2 at lower diagonal) for three SNPs at ABO locus. In our meta-GWAS, the SNP rs2519093 was found to be best associated with total campesterol when analysing an additive model. The SNP rs657152 was found in our previous GWAS when analysing a recessive model. This SNP is in perfect LD (with respect to both, D' and r^2) with rs8176719 representing the miss-sense mutation for the blood group allele O. Our new SNP rs2519093 is in perfect LD with these SNPs with respect to D' but not r^2. In line with this observation, a recessive model for rs2519093 can be assumed, also resulting in genome-wide significance.

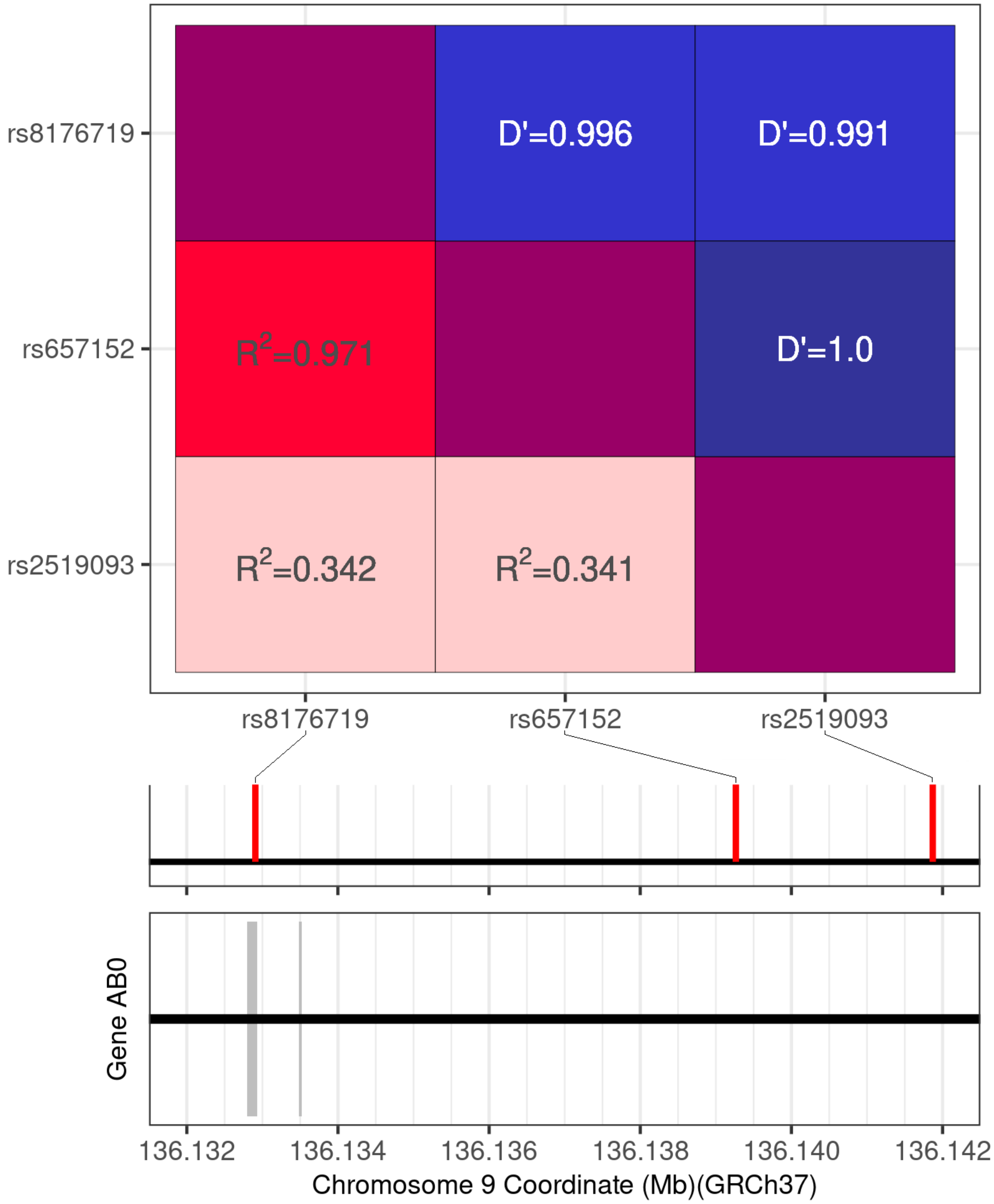

Supplementary Figure S8 (Mendelian Randomization Approach): Sketch of our Mendelian randomization approach. Genetic instruments of total sitosterol (G1) are used to determine causal effects on total cholesterol (α) and CAD (τ). Genetic instruments on total cholesterol (G2) are used to estimate the causal effect on CAD (β). We also split the causal effect of total sitosterol on CAD in a direct effect and an indirect effect mediated by total cholesterol (τ-α*β, respectively α*β).

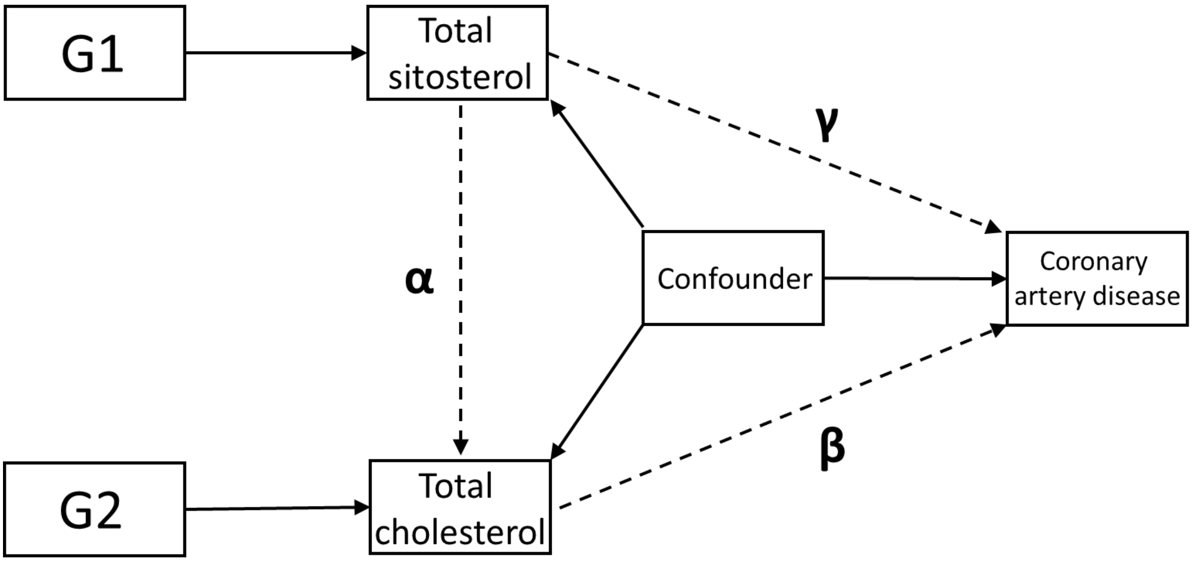

Supplementary Figure S9 (Results of Mendelian Randomization Analysis in Europeans): Panels on the left represent single instrumental effects on the axis traits. Crosses correspond to their respective 95% confidence intervals (beta estimates ± 1.96 * standard errors). In the right panels, results of different MR methods are displayed (IVW = inverse variance). Methods showed similar results throughout. A) causal effect of total cholesterol on CAD, B) causal effect of total sitosterol on CAD, C) causal effect of total sitosterol on total cholesterol, D) causal effect of normalized total sitosterol on CAD (analysed after excluding YFS due to heterogeneity). Numerical results are shown in supplemental data S14. Sample sizes for error bars are N=62,166 (TC), 122,733 cases / 424,528 controls (CAD), 9,755 (SIT) and 9,323 (SIT/CHT).

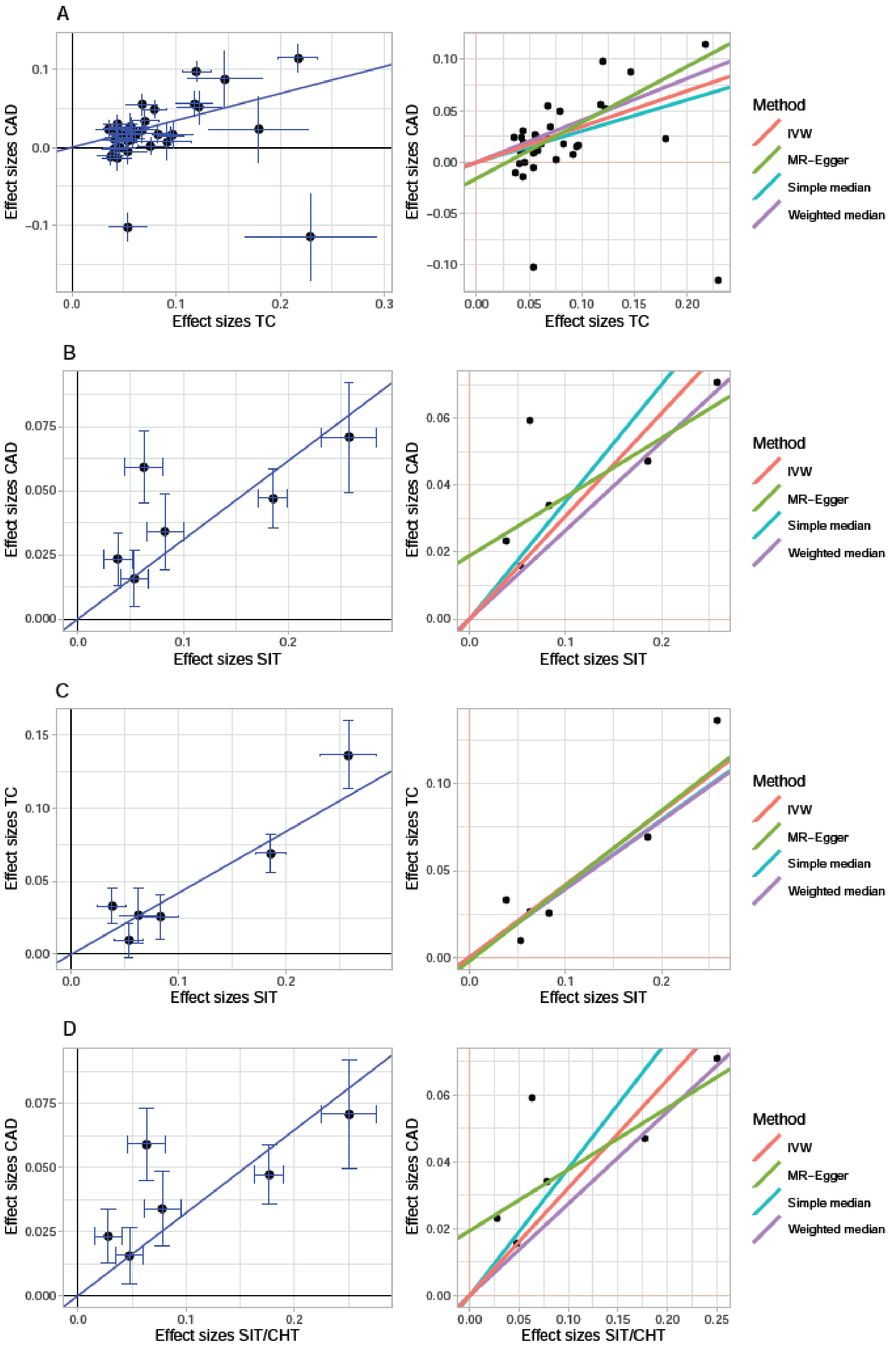

A
B
C
D
Effect sizes CAD
Effect sizes TC
Effect sizes SIT
Effect sizes SIT/CHT
Method
IVW
MR−Egger
Simple median
Weighted median

Supplementary Figure S10 (Results of Mendelian Randomization Analysis in Biobank Japan data): Panels on the left represent single instrumental effects on the axis traits. Crosses correspond to their respective 95% confidence intervals (beta estimates ± 1.96 * standard errors). In the right panels, results of different MR methods are displayed (IVW = inverse variance). Methods showed similar results throughout. A) causal effect of total cholesterol (TC) on coronary artery disease (CAD), B) causal effect of total sitosterol (SIT) on CAD, C) causal effect of total sitosterol on total cholesterol, D) causal effect of normalized total sitosterol (SIT/CHT) on CAD (analysed after excluding YFS due to heterogeneity). Numerical results are shown in supplemental data S15. Sample sizes for error bars are N=62,166 (TC), 25,892 cases and 142,336 controls (CAD), 9,755 (SIT) and 9,323 (SIT/CHT).

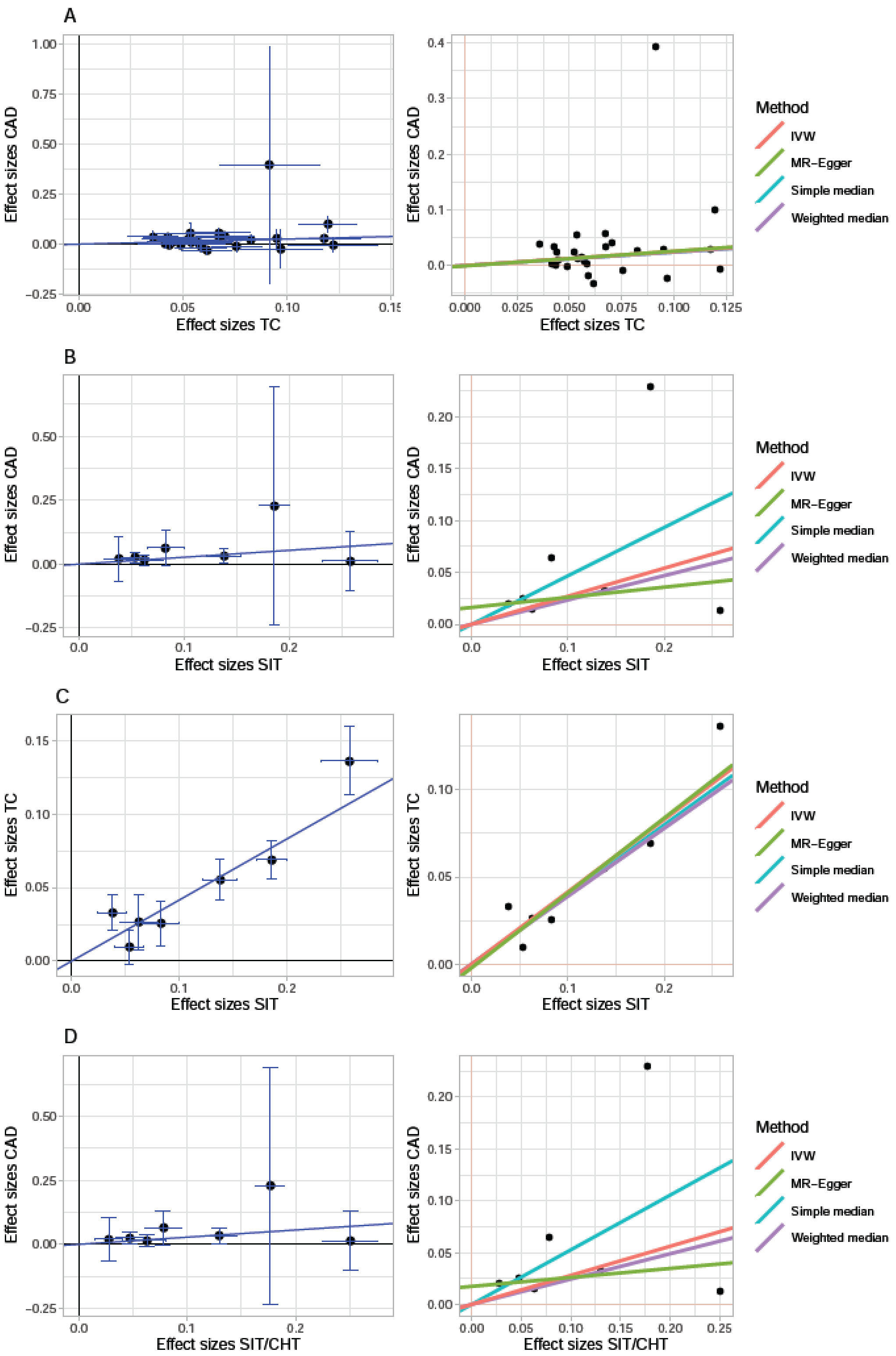
A
B
C
D
Effect sizes CAD
Effect sizes TC
Effect sizes SIT
Effect sizes SIT/CHT
Method
IVW
MR−Egger
Simple median
Weighted median

Supplementary Figure S11 (Study design): Summary of contributing studies, meta-analysis results, subsequent secondary analyses and major outcomes of these. A total of 32 phenotypes were studied comprising raw concentrations of free, esterified and total phytosterols. Free to esterified phytosterol ratios, and ratios of phytosterols to lanosterol and cholesterol, respectively. QC = quality controlled, eQTL = expression quantitative trait locus, CAD = coronary artery disease.

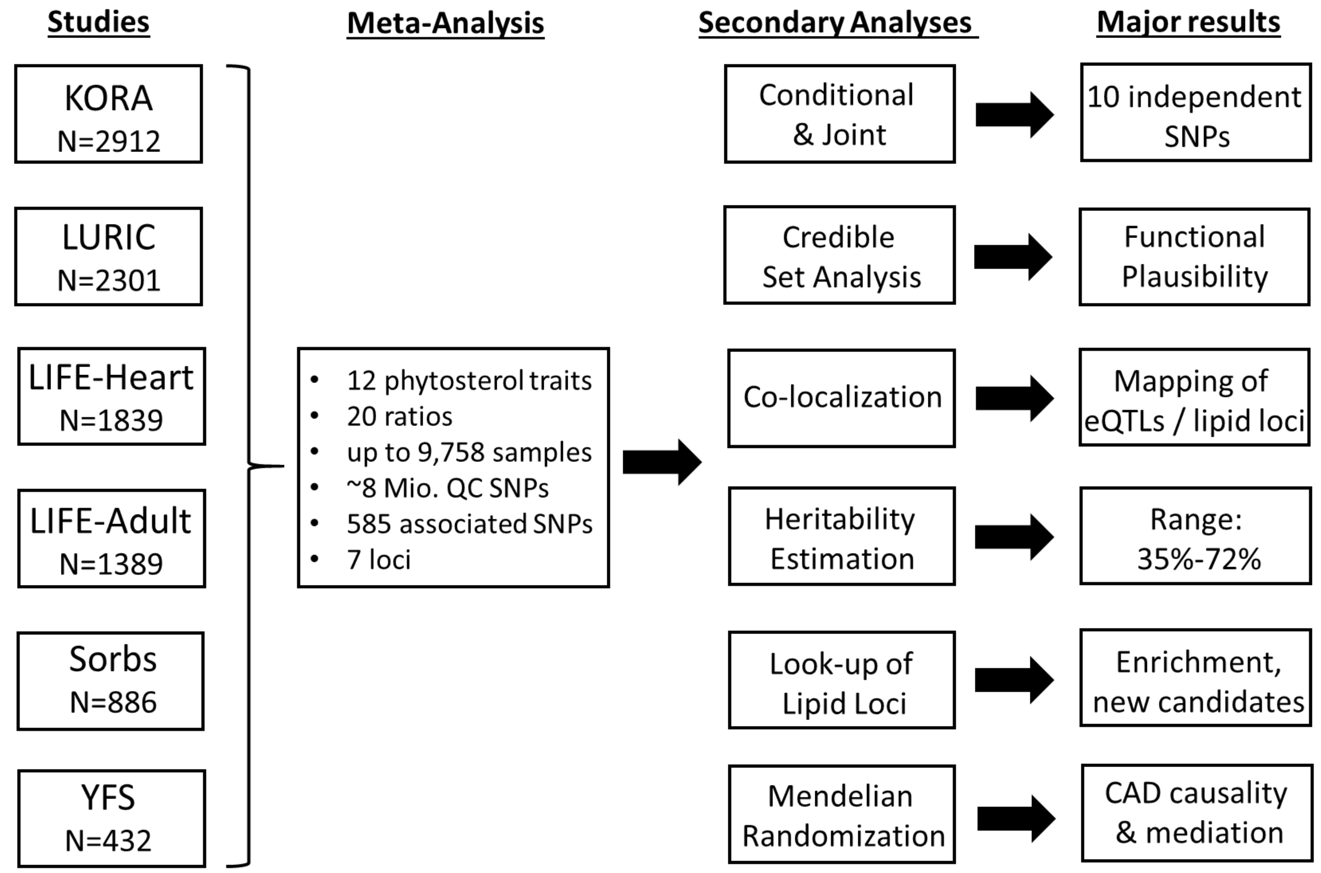

Supplement: Supplementary file 1 — Supplementary Information [file 41467_2021_27706_MOESM1_ESM.pdf]
