# Supplementary material for: Genome-wide meta-analysis of phytosterols reveals five novel loci and a detrimental effect on coronary atherosclerosis
Source: Nat Commun. 2022 Jan 10;13:143. doi: 10.1038/s41467-021-27706-6 (PMC8748632; doi:10.1038/s41467-021-27706-6)
Supplement: Supplementary file 3 — Description of Additional Supplementary Files [file 41467_2021_27706_MOESM3_ESM.pdf]

Description of Additional Supplementary Files

File Name: Supplementary Data 1
Description: Description of contributing studies

File Name: Supplementary Data 2
Description: Descriptive statistics of contributing studies and detailed information on genotyping, imputation and genetic data analysis

File Name: Supplementary Data 3
Description: Sample sizes and SNP numbers. We present sample sizes and SNP numbers after quality control per trait and study as well as those available for the genetic meta-analysis. Abbreviations of traits are provided in supplemental data set S15.

File Name: Supplementary Data 4
Description: Inflation factors. Per trait, we present genomic control derived inflation factors of meta-analysis results, for both, fixed effect and random effects models.

File Name: Supplementary Data 5
Description:
a: Annotation of genome-wide significant SNPs. We present statistics and annotations of all SNPs showing genome-wide significance in fixed effect meta-analysis for at least one of our traits. Columns are explained in supplemental data set S5c.
b: eQTL look-up of genome-wide significant SNPs. We present statistics and annotations of all eQTLs in linkage disequilibrium (r2>0.3) with our genome-wide significant SNPs. GTEx v7 and own data are used for this purpose. Columns are explained in supplemental data set S5c.
c: Explanation of columns of supplemental data sets 5a and b.

File Name: Supplementary Data 6
Description: Overview of association p-values for the ten identified independent SNPs across traits. We present -log10 of p-values.

File Name: Supplementary Data 7
Description: Best association p-value per identified locus and trait. We present -log10 of p-values.

File Name: Supplementary Data 8
Description:
a: Annotation of SNPs in 99% credible sets of independent variants. Columns are explained in supplemental data set S8c.
b: eQTL look-up of SNPs in credible sets of independent variants. Columns are explained in supplemental data set S8c.
c: Explanation of columns of supplemental data sets 8a and b.

File Name: Supplementary Data 9
Description:
a: Summary of colocalization results. We present the summary of identified colocalizations for each of the ten independent variants. Details are shown in supplemental data sets S9b-d. Association statistics correspond to fixed effect meta-analysis results of gene-dose effects of the best associated trait.

b: Colocalization results of the six loci with a single independent variant. We present numbers of SNPs used for the respective co-localization analysis and the posterior probabilities of the four hypotheses. Values larger than 0.8 are marked in red, while probabilities in between 0.6 and 0.8 are marked in yellow. Results are sorted by the posterior probability for H4 (evidence for co-localization)
c: Colocalization results of the four independent variants at the 2p21 locus. Conditional statistics were used for that purpose. We present numbers of SNPs used for the respective colocalization analysis and the posterior probabilities of the four hypotheses. Values larger than 0.8 are marked in red, while probabilities in between 0.6 and 0.8 are marked in yellow. Results are sorted by the posterior probability for H4 (evidence for co-localization)
d: Statistics of colocalization analyses. For each independent variant, we present association statistics (fixed effect meta-analysis of gene-dose effect) and colocalization results with total cholesterol, LDL-C, coronary artery disease and eQTLs of candidates in respective tissues. Beta = Beta estimator, SE = Standard error, pval = p-value, n = sample size, r2 = explained variance, coloc = posterior probability for H4 (evidence for co-localization)

File Name: Supplementary Data 10
Description: Genetic model at ABO locus. We perform detailed analysis of the genetic model at this locus considering total campesterol associations. First, by testing heterocygote deviations, we show that there is a significant deviation from the additive model used in overall genome-wide association analysis. Accordingly, the recessive reference allele model resulted in stronger effects and significances. Beta = Beta estimator, SE = Standard error, pval = p-value.

File Name: Supplementary Data 11
Description: Heritability estimates. We estimated heritabilities of all phytosterol traits in LIFE-Adult, LIFE-Heart and the meta-analysis of both using GCTA. N = sample size, h2 = heritability, SE h2 = standard error, p h2 = p-value of heritability estimate. Trait abbreviations are explained in supplemental data set S18.

File Name: Supplementary Data 12
Description:
a: GWAS lookup for known lipid SNPs - Summary. Total numbers of lipid SNPs and loci available in our data are shown. We provide numbers of SNPs and loci associated with phytosterol phenotypes (nominal significance: $p < 0.05$, suggestive significance: $p < 1e-6$, genome wide significance: $p < 5e-8$). Proportion of nominally significantly associated SNPs (rep_SNPs_p) respectively loci (rep_lovi_p) is provided. We also show the top associated phytosterol phenotype achieving genome-wide significance at lipid loci. Detailed statistics are provided in supplemental data set S12b.
b: GWAS lookup for known lipid SNPs - Detailed statistics. In columns E-H, we provide association p-values of lipid traits (total cholesterol, HDLC, LDLC and triglycerides) as reported in the literature. For each of these lipid SNPs, we provide detailed statistics of phytosterol associations available in our data. See supplemental data set 5c for an explanation of columns.

File Name: Supplementary Data 13
Description: Lookup of candidate genes for phytosterol esterification. We looked up variants in the genes LCAT, ACAT, SOAT1 and SOAT2 in order to search for variants associated with phenotypes of phytosterol esterification. See supplemental data set 5c for an explanation of columns.

File Name: Supplementary Data 14

Description: Mendelian Randomization analyses and sensitvity analyses in Europeans. We report results of different selections of instrumental variables. Scenario = analysed effect, beta_IVW, se_IVW, p_IWV = Mendelian Randomization Beta, standard error and p-value using inverse variance method, Q, p_Q = heterogeneity and p-value, nSNPs = number of instruments used, Prop dirEffect = Proportion of direct effect compered to total effect. Single variant statistics of total sitosterol and total cholesterol used for analyses are presented in supplemental data sets S16 and S17, respectively .

File Name: Supplementary Data 15
Description: Mendelian Randomization analyses and sensitvity analyses using CAD summary statistics from Japanese subjects. We report results of different selections of instrumental variables. Scenario = analysed effect, beta_IVW, se_IVW, p_IWV = Mendelian Randomization Beta, standard error and p-value using inverse variance method, Q, p_Q = heterogeneity and p-value, nSNPs = number of instruments used, Prop dirEffect = Proportion of direct effect compered to total effect. CAD summary statistics of Japanese subjects were used. Single variant statistics of total sitosterol and total cholesterol used for analyses are presented in supplemental data sets S16 and S17, respectively .

File Name: Supplementary Data 16
Description: Single variant statistics for total sitosterol instruments used for Mendelian Randomization analyses. We present the single variant statistics used for Mendelian Randomization analyses of supplemental data sets S14 and S15. In detail, we present association results of total sitosterol, total cholesterol, CAD in Europeans and CAD in Japanese for the instruments used in the analyses. "NA" is displayed if respective summary statistics are not available. For causal inference, only instruments with complete summary statistics are used. We also present allele frequencies for comparisons between European and Japanese ethnicities. Genome-wide significant associations are marked with red.

File Name: Supplementary Data 17
Description: Single variant statistics for total cholesterol instruments used for Mendelian Randomization analyses. We present the single variant statistics used for Mendelian Randomization analyses of supplemental data S14 and S15. In detail, we present association results of total sitosterol, total cholesterol, CAD in Europeans and CAD in Japanese for the instruments used in the analyses. "NA" is displayed if respective summary statistics are not available. For causal inference, only instruments with complete summary statistics are used. We also present allele frequencies for comparisons between European and Japanese ethnicities. Genome-wide significant associations are marked with red. Strong instruments for total cholesterol are marked with dark-red.

File Name: Supplementary Data 18
Description: Abbreviations for phytosterol traits used in tables.

File Name: Supplementary Data 19
Description: List of sofware versions used for analyses.
