# Supplementary material for: Genome-wide meta-analysis of phytosterols reveals five novel loci and a detrimental effect on coronary atherosclerosis
Source: Nat Commun. 2022 Jan 10;13:143. doi: 10.1038/s41467-021-27706-6 (PMC8748632; doi:10.1038/s41467-021-27706-6)
Supplement: Supplementary file 5 — Reporting Summary [file 41467_2021_27706_MOESM5_ESM.pdf]

nature research

Corresponding author(s): Markus Scholz

Last updated by author(s): Oct 25, 2021

Reporting Summary

Nature Research wishes to improve the reproducibility of the work that we publish. This form provides structure for consistency and transparency in reporting. For further information on Nature Research policies, see our Editorial Policies and the Editorial Policy Checklist.

Statistics

For all statistical analyses, confirm that the following items are present in the figure legend, table legend, main text, or Methods section.

n/a	Confirmed	
☐	☒	The exact sample size (*n*) for each experimental group/condition, given as a discrete number and unit of measurement
☐	☒	A statement on whether measurements were taken from distinct samples or whether the same sample was measured repeatedly
☐	☒	The statistical test(s) used AND whether they are one- or two-sided<br>*Only common tests should be described solely by name; describe more complex techniques in the Methods section.*
☐	☒	A description of all covariates tested
☐	☒	A description of any assumptions or corrections, such as tests of normality and adjustment for multiple comparisons
☐	☒	A full description of the statistical parameters including central tendency (e.g. means) or other basic estimates (e.g. regression coefficient) AND variation (e.g. standard deviation) or associated estimates of uncertainty (e.g. confidence intervals)
☐	☒	For null hypothesis testing, the test statistic (e.g. *F*, *t*, *r*) with confidence intervals, effect sizes, degrees of freedom and *P* value noted<br>*Give P values as exact values whenever suitable.*
☒	☐	For Bayesian analysis, information on the choice of priors and Markov chain Monte Carlo settings
☒	☐	For hierarchical and complex designs, identification of the appropriate level for tests and full reporting of outcomes
☐	☒	Estimates of effect sizes (e.g. Cohen's *d*, Pearson's *r*), indicating how they were calculated

Our web collection on statistics for biologists contains articles on many of the points above.

Software and code

Policy information about availability of computer code

Data collection	R 3.6.0
Data analysis	IMPUTE2, Plink 1.9, Plink 2.0, SNPTEST 2.5, GCTA version 1.92.0beta3, R 3.6.0 (all packages and versions are povided in supplemental data S19)

For manuscripts utilizing custom algorithms or software that are central to the research but not yet described in published literature, software must be made available to editors and reviewers. We strongly encourage code deposition in a community repository (e.g. GitHub). See the Nature Research guidelines for submitting code & software for further information.

Data

Policy information about availability of data

All manuscripts must include a data availability statement. This statement should provide the following information, where applicable:

- Accession codes, unique identifiers, or web links for publicly available datasets
- A list of figures that have associated raw data
- A description of any restrictions on data availability

Genome-wide summary statistics generated in this study have been deposited at https://doi.org/10.5281/zenodo.5607612. Used public data bases are: Deleteriousness scores (http://www.regulomedb.org/), GWAS catalogue (https://www.ebi.ac.uk/gwas/api/search/downloads/full), eQTLs (ftp://ftp.ncbi.nlm.nih.gov/eqtl/original_submissions/FHS_eQTL/). DOSE and Reactome pathways were retrieved via respective R-packages (see supplemental data S19). Genome-wide summary statistics of other studies were retrieved from web resources mentioned in the respective publications (see methods).

Field-specific reporting

Please select the one below that is the best fit for your research. If you are not sure, read the appropriate sections before making your selection.

☒ Life sciences ☐ Behavioural & social sciences ☐ Ecological, evolutionary & environmental sciences

For a reference copy of the document with all sections, see nature.com/documents/nr-reporting-summary-flat.pdf

Life sciences study design

All studies must disclose on these points even when the disclosure is negative.

Sample size	Due to the hypothesis-free nature of genome-wide association studies, we used the maximum number of available sample sizes of the traits of interest.
Data exclusions	All subjects with high-quality genotype information, available traits and covariables were used. No exclusions were performed.
Replication	We performed a meta-analysis of all available studies. Effect estimates of reported loci showed consistent effects accross studies.
Randomization	Not applicable. Only data from observational studies were considered. Covariate effects were considered during regression analysis.
Blinding	No group comparisons were performed. Blinding was not meaningful in our analysis context.

Reporting for specific materials, systems and methods

We require information from authors about some types of materials, experimental systems and methods used in many studies. Here, indicate whether each material, system or method listed is relevant to your study. If you are not sure if a list item applies to your research, read the appropriate section before selecting a response.

Materials & experimental systems

n/a	Involved in the study	
☒	☐	Antibodies
☒	☐	Eukaryotic cell lines
☒	☐	Palaeontology and archaeology
☒	☐	Animals and other organisms
☐	☒	Human research participants
☒	☐	Clinical data
☒	☐	Dual use research of concern

Methods

n/a	Involved in the study	
☒	☐	ChIP-seq
☒	☐	Flow cytometry
☒	☐	MRI-based neuroimaging

Human research participants

Policy information about studies involving human research participants

Population characteristics	KORA participants (N=2928) have an average age of 47 years with 52% females. LIFE-Adult participants (N=1389) have an average age of 70 years with 48% females. LIFE-Heart participants (N=1839, N=762 with CAD) have an average age of 63 years with 36% females. LURIC participants (N=2300, N=1567 with CAD) have an average age of 63 years with 31% females. Sorbs participants (N=886) have an average age of 48 years with 59% females. YFS participants (N=432) where males throughout with an average age of 36. years. Further descriptive statistics are provided in supplemental data S2.
Recruitment	KORA and LIFE-Adult are age- and sex-stratified population-based cohorts from Germany. LIFE-Heart and LURIC are patients with suspected or confirmed coronary artery disease. The Sorb study is a convenience sample of members of the self-contained Sorb population in Germany. YFS is a cohort study from Finland. Study details can be found in the supplement material S1. We do not expect a bias in the meta-analysis results due to low selective pressure of the phenotypes of interest. Only variants in Hardy-Weinberg equilibrium and low heterogeneity of association results accross studies were reported.
Ethics oversight	KORA was approved by the Ethics Committee of the Bavarian Medical Association. LIFE-Adult, LIFE-Heart and the Sorbs study were approved by the University of Leipzig's ethics committee (Reg.-No. 263-2009-14122009, 276/05-ek, 088-2005). LURIC was approved by the institutional review board of the ethics committee of the Landesärztekammer Rheinland-Pfalz (Reg.-No. 1997-203). YFS was approved by the local ethics committee of the Hospital District of Southwest Finland and the regional Ethics Committee of the Expert Responsibility area of Tampere University Hospital, Finland.

Note that full information on the approval of the study protocol must also be provided in the manuscript.
